# Hyaluronan, Inflammation, and Breast Cancer Progression

**DOI:** 10.3389/fimmu.2015.00236

**Published:** 2015-06-08

**Authors:** Kathryn L. Schwertfeger, Mary K. Cowman, Patrick G. Telmer, Eva A. Turley, James B. McCarthy

**Affiliations:** ^1^Department of Laboratory Medicine and Pathology, Masonic Comprehensive Cancer Center, University of Minnesota, Minneapolis, MN, USA; ^2^Biomatrix Research Center, Department of Chemical and Biomolecular Engineering, New York University Polytechnic School of Engineering, New York, NY, USA; ^3^Department of Oncology, London Health Science Center, Schulich School of Medicine, Western University, London, ON, Canada; ^4^Department of Biochemistry and Surgery, London Health Science Center, Schulich School of Medicine, Western University, London, ON, Canada

**Keywords:** hyaluronan, breast cancer, inflammation, tumor microenvironment, RHAMM/HMMR, CD44, macrophage

## Abstract

Breast cancer-induced inflammation in the tumor reactive stroma supports invasion and malignant progression and is contributed to by a variety of host cells including macrophages and fibroblasts. Inflammation appears to be initiated by tumor cells and surrounding host fibroblasts that secrete pro-inflammatory cytokines and chemokines and remodel the extracellular matrix (ECM) to create a pro-inflammatory “cancerized” or tumor reactive microenvironment that supports tumor expansion and invasion. The tissue polysaccharide hyaluronan (HA) is an example of an ECM component within the cancerized microenvironment that promotes breast cancer progression. Like many ECM molecules, the function of native high-molecular weight HA is altered by fragmentation, which is promoted by oxygen/nitrogen free radicals and release of hyaluronidases within the tumor microenvironment. HA fragments are pro-inflammatory and activate signaling pathways that promote survival, migration, and invasion within both tumor and host cells through binding to HA receptors such as CD44 and RHAMM/HMMR. In breast cancer, elevated HA in the peri-tumor stroma and increased HA receptor expression are prognostic for poor outcome and are associated with disease recurrence. This review addresses the critical issues regarding tumor-induced inflammation and its role in breast cancer progression focusing specifically on the changes in HA metabolism within tumor reactive stroma as a key factor in malignant progression.

## Hyaluronan as a Component of Cancerized Stroma in Human Breast Cancer

During breast cancer growth, tumor cells interact with their surrounding stroma to create an environment resembling that found during wound healing with increased inflammation, angiogenesis, and stromal remodeling. Both tumor cells and fibroblasts produce pro-inflammatory chemokines and cytokines, which recruit and activate innate immune cells including neutrophils and macrophages (1). Macrophages are recruited by tumor cells as a key component of the inflammatory microenvironment and have been strongly implicated in breast cancer growth and progression in patients (1–4). Together, the tumor cells, fibroblasts, and inflammatory cells produce factors that remodel the extracellular matrix (ECM), leading to the formation of a “cancerized” microenvironment that sustains tumor growth and promotes malignant progression. The ECM is composed of proteins and proteoglycans/glycosaminoglycans that provide structural support and facilitate tissue organization. In addition, specific components of the ECM contribute to cell survival, proliferation, migration, angiogenesis, and immune cell infiltration. A major ECM component of the stroma is hyaluronan (HA), a member of the glycosaminoglycan family of polysaccharides. HA is synthesized at the cell surface as a large linear anionic polymer (up to 10^7^ Da) by multiple cell types in healing wounds and in tumors. There are three distinct isoenzymes (HA synthases, HAS 1–3) that synthesize HA (see below). Understanding the role that HA plays in contributing to breast carcinoma-induced inflammation has important implications for the design of therapeutic approaches targeting both the tumor cells and the pro-tumorigenic functions of the cancerized stroma (5).

A number of studies have demonstrated that HA regulates tumor cell migration and invasion *in vitro*, and tumor growth and progression *in vivo* (5–7). Cell culture studies show that invasive breast cancer cells synthesize and accumulate larger amounts of HA than normal tissue and preferentially express more HAS2 mRNA than less aggressive tumor cells (8). Furthermore, HAS2 promotes breast cancer cell invasion *in vitro* (9). Overexpression of HAS2 in mammary epithelial cells of MMTV-Neu transgenic mice increases tumor HA production and enhances growth of mammary tumors (10). HAS2-overexpressing tumors exhibit enhanced angiogenesis and stromal cell recruitment. These results demonstrate that increased HA in the tumor microenvironment supports mechanisms of neoplastic progression.

Although carcinoma cells synthesize HA, stromal HA levels are increased in breast cancers predicting that stromal cells are also a rich source of this biopolymer. Similar to the contributions of HA fragments in wound healing, HA fragmentation leads to the generation of angiogenic fragments that act on endothelial cells to promote blood vessel formation (11). As described below, HA regulates inflammatory cell functions located within the tumor microenvironment. The combined effects of HA on both tumor and host cells as well as evidence that elevated accumulation of peri-tumor stromal HA is linked to reduced 5-year survival (12), provide strong evidence that HA participates in the generation of a pro-tumorigenic “cancerized” stroma (5).

In-depth analysis of the HA staining patterns within tumors shows an enrichment of HA in the stroma at the leading edge of the tumor, and detailed clinical study of these HA levels and localization in patient samples support a relationship between high-stromal HA accumulation and poor patient survival (12). While most HA is likely synthesized by stromal cells, a subset of breast cancers also stain for HA in the tumor parenchyma and this is correlated with lymph node positivity and poor differentiation (12). Furthermore, these tumors tend to be negative for the hormone receptors estrogen receptor (ER) and progesterone receptor (PR) (12).

Hyaluronan accumulation has additionally been compared in early and later stage breast tumors, specifically in ductal carcinoma *in situ* (DCIS), DCIS with microinvasion and invasive carcinoma, to determine if altered HA production is linked to early as well as later stage invasion events in breast cancer. HA levels of DCIS associated with microinvasion and later stage invasive carcinoma are significantly increased when compared to pure DCIS (13). RHAMM/HMMR, which promotes migration and invasion of breast cancer lines, is also elevated in breast cancer, particularly at the invasive front of tumors and in tumor cell subsets (14, 15). Collectively, these results suggest that HA performs a number of functions in progressing tumors and in particular contributes to invasion in early and later stage breast cancer.

More recently, HA staining and CD44 expression have been examined in HER2-positive breast tumors. High levels of stromal HA staining in this breast cancer subtype have been linked to specific clinical correlates, including lymph node-positive breast cancer and reduced overall survival (16). Elevated CD44 expression, which occurs in tumor parenchyma and to a lesser extent in stromal cells, is associated with HER2-positive breast cancers and linked to reduced overall survival in this breast cancer subtype. A number of studies have also examined expression levels of HA synthases in breast cancer tissues. Expression of all of the HAS isoenzymes (HAS 1–3) have been detected in the tumor parenchyma and stroma of breast tumors (17). Expression of tumor cell HAS1, but not HAS2 or HAS3, was found to correlate with reduced overall survival when breast cancer patients were not sorted into subtypes. In this study, expression of all three HAS proteins in the stroma corresponds with reduced overall survival (17). However, HAS2 expression is particularly linked to triple negative and basal-like breast cancer subtypes and its elevated expression is associated with reduced overall survival of these cancer patients (18).

Together, these studies suggest pro-tumorigenic roles for increased levels of HA in breast cancer (19, 20) and predict possible mechanisms through which HA might facilitate tumor initiation and progression. For example, the increase in tumor cell HA may provide a self-protective coat, minimizing recognition by immune cells and helping to reduce damage by reactive oxygen and nitrogen species. Increased levels of HA may also facilitate mitosis and invasion of surrounding tissue. However, recent studies demonstrating that fragmentation of HA within damaged tissues alters biological properties of the intact biopolymer and that HA receptors differ in their recognition of HA polymer sizes suggest a much more complex mode of regulation (see below). These more recent studies emphasize the importance of defining both changes in HA levels and the extent of HA fragmentation for understanding mechanisms by which the peri-tumor stroma, in particular the inflammatory status of the tumor-associated stroma, influences breast cancer initiation and progression.

## Regulation of HA Synthesis and Fragmentation

**Figure 1:**
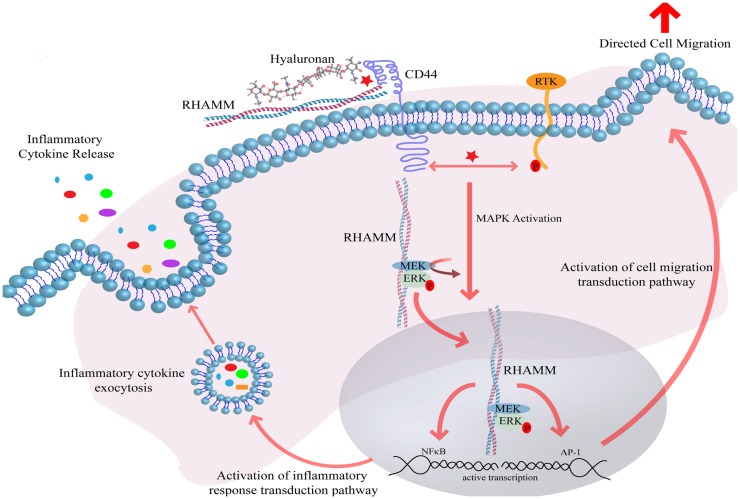
**Hyaluronan induces receptor-mediated signaling through interaction with cell-surface HA binding proteins**. Interaction of HA with CD44 and RHAMM induces CD44 receptor clustering and intracellular RHAMM-regulated MAPK activation, resulting in ERK phosphorylation and downstream activation of the transcription effectors AP-1 and NFκB. Active transcription of AP-1 and NFκB target genes ultimately result in the induction of directed cell migration and release of inflammatory cytokines. Abbreviations: MEK, MAPK ERK kinase; ERK, extracellular regulated kinase; RHAMM, receptor for hyaluronan-mediated motility; CD44, cluster of differentiation 44; AP-1, activator protein 1; RTK, receptor tyrosine kinase; NFκB, nuclear factor kappa B.

Elevated HA synthesis in adults is most often associated with a response to tissue damage or disease. These increases result from both transcriptional and post-transcriptional control of HA synthesis. HA synthesis is catalyzed by one or more of three HA synthase isoenzymes (HAS 1–3) (21, 22), which are unique among other glycosyltransfererases since they are localized at the plasma membrane rather than in the Golgi (23). Primary structures of all three enzymes predict that they span the plasma membrane several times (21). The three isoenzymes contain cytoplasmic catalytic sites that sequentially add the activated UDP-d-glucuronic acid and UDP-*N*-acetyl-d-glucosamine to the growing HA polymer, which is then extruded through pores in the plasma membrane, likely created by formation of HAS oligomers (21, 22). Released HA polymers are captured by extracellular HA binding proteoglycans such as versican, along with other ECM protein components and cell-surface receptors (5, 6, 24). High-molecular weight polymers of HA are thought to function like other ECM components, in part, by providing a multivalent template to organize ECM proteoglycans and to cluster HA receptors thus “organizing” plasma membrane components. Clustering leads to subsequent cytoskeletal re-organization, the efficient assembly and activation of signaling pathways and ultimately changes in the cellular transcriptome (Figure 1).

Hyaluronan synthesis is controlled by multiple distinct but overlapping mechanisms. HA synthesis is partially regulated by intracellular levels of the HAS substrates UDP-GlcA and UDP-GlcNAc. These are produced by complex pathways that control their levels and/or availability within cells. For example, the compound 4-methyl-umbelliferone (4-MU) inhibits the synthesis of HA by depleting cytoplasmic UDP-GlcA, (25). Although 4-MU theoretically could limit substrate availability to multiple glycosyltransferases, its inhibitory effect appears to be localized to limiting substrate availability for HAS isoenzymes associated with the inner plasma membrane. By contrast, the majority of glycosyltransferases are resistant to the inhibitory effects of 4-MU since they are located in the Golgi, which is not permeable to 4-MU (23). The genes encoding HAS isoenzymes are located on distinct chromosomes and their expression is regulated by distinct transcriptional and post-transcriptional mechanisms (23, 26, 27). Numerous wound and tumor-associated cytokines and growth factors promote HA synthesis, including TGFβ, PDGF, FGF2, EGF, and TNFα [Ref. (23) and references therein]. It is important to emphasize that regulation of HAS isozyme expression and activity can be cell and tissue specific. Thus, careful analysis is needed when considering HA-related mechanisms in different pathologies. For example, HAS2 transcription can be regulated via EGFR/STAT3 pathways, PKA/CREB pathways (23), or TNFα or IL-1β-induced activation of NF-κB. The latter pathway is particularly relevant to the impact of up-regulated HA synthesis in the context of inflammation. While transcriptional control is a major mechanism for regulating HA synthesis, post-translational modification of HAS isoenzymes also affects their activity and/or cell-surface localization. For example, ErbB2/ERK1, 2 signaling activates and phosphorylates the three HAS isoenzymes implicating this as a mechanism for up-regulated HA synthesis in HER2/neu-positive tumor subtypes (28). HAS proteins can also be covalently modified by O-GlcNAcylation, which modifies trafficking and/or subcellular localization of the enzymes to the plasma membrane (23). Finally, early evidence from analyzing the naked mole rat genome shows that activating mutations of HAS2 can be selected for, that increase not only the production but also the predominant size of HA synthesized by this HAS isoform (29).

Large, native HA polymers clearly participate in the architectural maintenance and hydration of homeostatic adult tissues. However, recent evidence demonstrates that HA fragmentation is a critical contributing factor in the physiology of wounds and cancerized stroma (27, 30, 31). While larger HA polymers appear to be anti-inflammatory and anti-tumorigenic, HA fragments and oligomers are pro-inflammatory and pro-tumorigenic. This has led to the concept that HA fragmentation is one of the initial “danger signals” sensed by cells to initiate efforts that limit tissue damage through promoting tissue inflammation and repair (5). This cycle of increased synthesis and fragmentation appears to be hijacked by tumor cells and their stromal partners to sustain inflammation, which contributes to malignant progression. The mechanisms by which HA fragmentation contributes to such tissue pathology are not well understood. One proposed function is that LMW fragments alter or disrupt the cellular “organizing” properties of HMW HA by inhibiting the HA-induced clustering of cell-surface receptors such as CD44 and affecting signaling (6, 24, 32). Direct pull down assays of cellular extracts using beads coupled to HA oligomers have demonstrated that tumor cell and wound RHAMM can bind LMW HA fragments (33). This scenario predicts that cell-surface RHAMM, displayed in response to cellular stress, is one HA receptor that “senses” HA fragmentation and thus serves to initiate cellular responses to tissue damage possibly by affecting CD44 clustering (5). These previous studies point to the importance in determining both the level of HA and the ratio of HMW HA to LMW fragments noted previously (34, 35) and in an accompanying manuscript in this issue (36).

Hyaluronan fragmentation within tissues results from the increased expression of one or more hyaluronidases (Hyals) and from oxidative/nitrosative damage. Hyals function as endo- or exoglycosidases to cleave HA polymers (27, 30). Hyal1 and Hyal2 are most often associated with damaged or tumor-associated stroma undergoing remodeling (27). *In vitro* analysis of hyaluronidases indicates that their activity results in unique fragmentation patterns. For example, although both Hyal1 and Hyal2 can catalyze degradation by cleaving β-(1,4) linkages, they differ in that Hyal1 degrades HA into small fragments (hexasaccharides and tetrasaccharides) whereas Hyal2 appears to produce predominantly larger (i.e., 20 kDa) fragments (37). Both Hyal1 and Hyal2 have pH optima in the acidic range and are associated with processing HA that has been internalized into endocytic vesicles. However, low pH within localized stromal microenvironments facilitates extracellular Hyal-mediated HA degradation (27).

Hyaluronan is also fragmented by reactive oxygen and nitrogen species (ROS/RNS) such as hydroxyl radicals (•OH), peroxynitrite/peroxynitrous acid (ONOO^−^/ONOOH), and hypochlorite anion (OCl^−^). Iron, derived from tissue associated heme or ferritin, is one important contributor in catalyzing the formation of both hydroxyl radicals and superoxide anions ${\text{O}}_{2}^{-•}$. This mechanism is contributed to by infiltrating polymorphonuclear leukocytes, monocytes, and activated macrophages (38). HA is extensively cleaved by any of these reactive species, and they are therefore important mechanisms for HA fragmentation within inflamed tissues. Although assessment of HA fragmentation by these mechanisms have largely been defined using *in vitro* analyses (39), it is clear that this degree of HA fragmentation occurs in skin wound tissue and in human milk (34, 35).

## Cellular Receptors for Hyaluronan

Although a number of HA receptors have been identified, the two that have been best characterized and are to date most relevant to inflammation and breast cancer are CD44 and RHAMM (5, 27). Other receptors implicated in cellular responses to HA, TLR2, and TLR4, are discussed in more detail below. Interactions between HA and CD44 lead to ligand-induced clustering, and activation of intracellular signaling pathways such as ERK1, 2, Akt, and FAK. The binding of HA by CD44 occurs through interactions with an amino terminal “link” domain, similar to those found in several other types of HA binding proteins, in particular, extracellular HA binding proteoglycans such as versican, aggrecan, and link protein. RHAMM binds HA through structurally distinct domains (BX_7_B motifs where B is a basic amino acid residue and X are non-acidic residues) that differ from link domains (5, 27). While CD44 expression is ubiquitous, RHAMM is normally not detected in most homeostatic tissues, but expression increases in response to injury and thus seems to be primarily important for restoring homeostasis following injury (5). Null RHAMM mice are viable but exhibit defects in tissue response to injury including vascular damage and excisional wound healing (40). RHAMM may also be required for robust female fertility in mice (41). Interaction of HA with CD44 is often associated with increased cell motility and invasion, although numerous reports have demonstrated that CD44 can also modify growth and therapeutic resistance of tumor cells (6, 24). As with CD44, RHAMM is displayed on cell surfaces. However, unlike CD44, RHAMM surface expression is tightly regulated, occurring under conditions of cellular stress. Thus, RHAMM is largely a cytoplasmic protein whose surface localization is regulated by mechanisms similar to other non-conventionally exported cytoplasmic and nuclear proteins and that regulates signaling cascade activation through co-receptor functions with integral receptors such as CD44 (5). Cell surface and intracellular RHAMM are also involved in stimulating cell motility and invasion. Intracellular RHAMM co-distributes with interphase microtubules and a splice variant of human RHAMM has been detected in nuclei (14, 42). RHAMM expression increases in G2/M of the cell cycle, associating it with mitosis and modifying cell-surface RHAMM blocks cells in G2M (43). This is consistent with more recent reports indicating that RHAMM is a critical contributor to mitotic spindle formation and regulation of proper chromosomal segregation and genomic stability (44). Both CD44 and cell-surface RHAMM also function as co-receptors for activating transmembrane tyrosine kinases (including EGFR, c-MET, and PDGFR) and ERK1,2 (Figure 1).

Both CD44 and RHAMM regulate the intensity and/or duration of such signal transduction pathways as ERK1, 2, which are initiated by growth factors (40, 45). Intracellular RHAMM functions as a scaffold protein that directly binds to ERK1 and forms complexes with ERK1, 2, and MEK1. This has been proposed to be one mechanism by which RHAMM helps to increase the intensity and/or duration of oncogenic ERK1, 2 signaling pathways (46, 47). One consequence of HA, CD44, RHAMM-mediated increases in the duration of ERK 1, 2 activation is the alteration of the transcriptome of cells within the cancerized stroma (Figure 1). These changes in gene expression have an impact on the activation of transduction pathways related to cell migration and the expression and export of inflammatory mediators. In turn, the persistent activation of these pathways in cancerized stroma enhances pro-tumorigenic inflammation and breast tumor progression. Thus, this represents one major mechanism by which biological “information” encoded within HA can lead to pro-tumorigenic or “cancerized” alterations in stroma.

Positive paracrine and autocrine feedback loops between tumor and stromal cells can be initiated by inflammatory mediators such as IL-1α and TGFβ that increase HA synthesis and expression of both RHAMM and CD44, which collectively sustain cell migration and invasion within cancerized stroma. Thus, the aberrant upregulation of CD44 or RHAMM in cancerized stroma is a nefarious consequence of sustained ERK 1, 2 activation, further aggravating persistent oncogenic signaling (46, 47). Since CD44 and RHAMM functionally cooperate under certain conditions (40), targeting RHAMM may be an effective way to specifically limit the function of CD44 in breast tumors.

LYVE-1, another cell-surface HA receptor associated with cancerized stroma (48–50), was first identified as a surface marker expressed by lymphatic endothelium and has been proposed to serve in HA transport from interstitial tissue to lymph (51). However, studies addressing the obligatory importance of LYVE-1 in promoting normal lymphangiogenesis have yielded conflicting results (52, 53). In tumors, the density of stromal LYVE-1 positive lymphatic vessels is a negative prognostic indicator in breast cancer patients with invasive ductal carcinomas (54). Furthermore, *in vitro* studies suggest that HA and LYVE-1 promote adhesion of breast cancer cells to fibroblasts, predicting these interactions contribute to adhesion or dissemination of tumor cells (55). Nevertheless, a mechanistic role for LYVE-1 in poor prognosis of breast cancer has yet to be demonstrated. One possible mechanism is suggested by the expression of LYVE-1 in cancer-associated macrophages (56) but a causative role for this HA receptor in inflammation has yet to be established.

## Effects of Hyaluronan on Innate Immune Cells in Cancerized Stroma

The generation of a pro-tumorigenic inflammatory environment during breast cancer initiation and progression requires recruitment of inflammatory cells, including neutrophils and macrophages. Once recruited to the tumor site, these cells become activated and secrete factors that are normally involved in proliferation, angiogenesis, and stromal remodeling during tissue repair (1). Macrophages residing within the tumor parenchyma and the tumor reactive stroma are prognostic of poor outcome in breast cancer patients (57). Macrophages in a wound-healing context are characterized as pro-inflammatory (M1) or anti-inflammatory (M2) (58). Pro-inflammatory macrophages are involved in the initial stages of wound healing and are characterized by the expression of NF-κB-regulated pro-inflammatory cytokines, including IL-1β and IL-12 as well as mediators contributing to pathogen destruction, including reactive oxygen species. Anti-inflammatory macrophages are important for the resolution phase of the wound-healing process and they are characterized by expression of anti-inflammatory cytokines, including TGFβ and IL-10 as well as factors that promote tissue remodeling including the MMPs. Profiling and functional studies demonstrate that macrophages within the tumor microenvironment express a range of both pro- and anti-inflammatory factors depending upon tumor type and stage. For example, macrophages associated with early stages of tumorigenesis have high levels of NF-κB activation and subsequently express pro-inflammatory factors, such as IL-1β and IL-6 (59). As tumors become increasingly aggressive, tumor-associated macrophages express high levels of immunosuppressive cytokines, such as IL-10 and TGFβ (58). Tumor-associated macrophages also produce factors that are established promoters of breast cancer growth and progression including EGF, VEGF, and MMP-9 (60). Thus, it is clear that tumor-associated macrophages reside in a functional continuum that is regulated by specific factors within the tumor microenvironment. However, the specific factors within the microenvironment that macrophages are responding to and driving these responses are not well understood.

A primary function of monocytes and macrophages in wound-healing environments is to produce reactive oxygen intermediates, which contribute to pathogen killing during wound healing (58). High levels of reactive oxygen species, found in both wound healing and tumor environments, are known to fragment HA, which then induce expression of pro-inflammatory genes (38, 61, 62). Recent studies of human breast cancer samples demonstrate that high numbers of CD163 positive macrophages correlate with increased levels of HA synthases and HA accumulation within tumors (63). Based on the links between HA and macrophages during wound healing, it is likely that HA in the tumor microenvironment may regulate macrophage function.

Indeed, HA modulates expression levels of pro-tumorigenic cytokines and chemokines in macrophages. Specifically, HA induces expression of the pro-inflammatory cytokine IL-1β in macrophages (64). Numerous studies have implicated IL-1β in breast cancer initiation and progression. Expression of IL-1β is increased in tumor and stromal cells in 90% of ER negative invasive breast carcinomas (65, 66). In addition, high levels of serum IL-1β correlate with recurrence in breast cancer patients (67). Finally, IL-1β may also be involved in premalignant breast cancer based on studies that showing increased IL-1β expression in pre-invasive DCIS (65, 68). Mechanistically, increased IL-1β within the tumor microenvironment leads to enhanced expression of cyclooxygenase-2 (COX-2), which contributes to the formation of early stage lesions and is a well-established tumor promoter (69). Increased IL-1β also leads to mammary tumor growth and metastasis in part through inducing regulation of myeloid derived suppressor cells (MDSCs), which promote an immunosuppressive environment (70). Taken together, these studies suggest that modulation of pro-inflammatory cytokines by HA in the tumor microenvironment represents a potential mechanism through which HA might contribute to tumor growth and progression.

**Figure 2:**
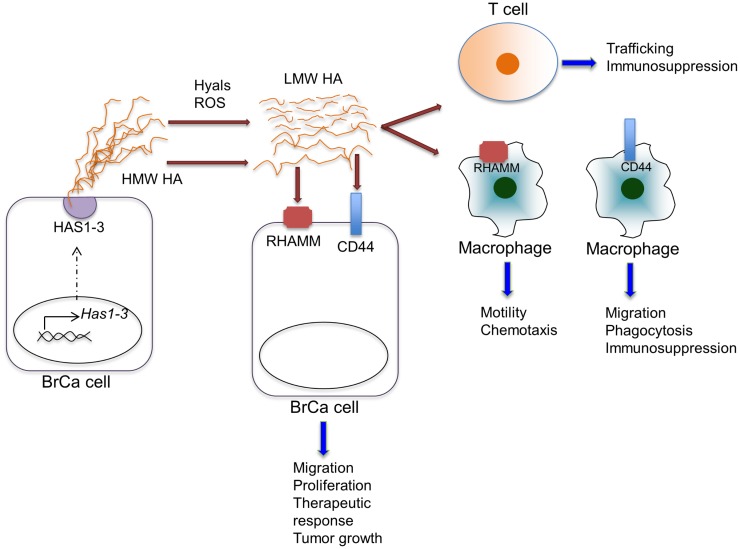
**Effects of HA on various cell types within the tumor microenvironment**. HA is synthesized by HAS enzymes in breast cancer cells (shown) or in stromal cells (not shown) and extruded to the extracellular space. HA can be fragmented by hyaluronidases (Hyal) or reactive oxygen species within the tumor microenvironment. HA, full length and fragmented, can act through cell-surface receptors, including CD44 and RHAMM, on various cell types to regulate the indicated cell functions.

The precise mechanisms by which elevated levels of stromal HA modulate pro-inflammatory responses are not well understood. Similar to the wound-healing environment, both increased levels of hyaluronidases (71, 72) and reactive oxygen or nitrogen species, including nitric oxide are present in breast tumors (39, 73), predicting elevated HA fragmentation in the tumor microenvironment. *In vitro* studies demonstrate that increased HA fragmentation is correlated with elevated hyaluronidase expression by breast cancer cells (74). Studies focusing specifically on hyaluronidase 1 (Hyal1) demonstrate that enhanced expression of Hyal1 in breast cancer cells induces tumor cell proliferation, migration, invasion, and angiogenesis (75). Furthermore, knock-down of Hyal1 in breast cancer cells reduces cell growth, adhesion, and invasion in culture as well as decreased tumor growth *in vivo* (72). Breast cancer cells lacking ER expression typically produce more hyaluronidases than estrogen positive cells and this correlates with invasion *in vitro* (15). LMW HA fragments, but not total HA levels, detected in the serum of breast cancer patients also correlates with the presence of lymph node metastasis (74). In addition, Hyal1 expression in non-invasive ductal hyperplasias correlates with subsequent development of invasive breast carcinoma (76). These studies indirectly establish a link between breast cancer and HA fragmentation (Figure 2), although studies analyzing the accumulation of HA fragments in experimental or clinical breast cancer tissues are still lacking. Because HA fragments are pro-inflammatory, it is reasonable to assume that they contribute to production of inflammatory cytokines, chemokines, and proteases by tumor-associated macrophages (27). In contrast to LMW fragments, HMW HA suppresses expression of many of the above pro-inflammatory cytokines in macrophages (77). This opposing function of native HA suggests that both the level and the distribution ratio of different size HA fragments may dictate inflammatory cell phenotypes within cancerized stroma. Development of new technologies to isolate and characterize HA polymers and fragments from tissues will be key for developing a mechanistic understanding of the biological complexities associated with HA metabolism (35).

In addition to regulating pro-inflammatory cytokine production, HA can modulate the expression of anti-inflammatory cytokines. Analysis of macrophage responses to tumor cell conditioned media demonstrates that tumor cell-derived HA stimulates production of IL-10 by macrophages (78). IL-10, an anti-inflammatory cytokine, is a potent mediator of immunosuppression in the tumor microenvironment through inhibition of T cell activation (79). Recent studies have demonstrated that increased IL-10 in the breast cancer microenvironment leads to therapeutic resistance through multiple potential mechanisms. For example, increased levels of IL-10 lead to the suppression of CD8^+^ T cell responses in response to chemotherapy (Figure 2) (4). Furthermore, IL-10 has been found to act directly on breast cancer cells to promote survival in response to chemotherapy involving a STAT3/bcl-2 mechanism (80). Thus, it is possible that HA contributes to immunosuppression and therapeutic resistance through modulation of IL-10 in the tumor microenvironment.

Hyaluronan also controls expression of chemokines, including IL-8/CXCL8 (81). Chemokines are pro-inflammatory cytokines that play an essential role in leukocyte recruitment and cell trafficking. These secreted proteins interact with cell-surface G-protein-coupled receptors to induce cytoskeletal rearrangement, adhesion to endothelial cells, and directional migration of cells to specific tissue sites (82). For example, IL-8 binds its receptors, CXCR1 and CXCR2, to stimulate neutrophil chemotaxis (67). IL-8 is overexpressed in breast cancers and contributes to tumor initiation and growth through promoting migration and invasion of breast cancer cells. More recently, studies have implicated IL-8 in the regulation of breast cancer stem cell invasion (83). Macrophage chemokines that are regulated by HA, including CXCL2 and CXCL12, have similarly been implicated in breast cancer progression (27) and have been shown to promote migration and invasion of these cancer cells (84, 85). The CXCL12/CXCR4 axis is particularly important for homing of breast cancer cells to metastatic sites, including bone and lung (86).

In another positive feedback loop, HA production is also modulated by pro-inflammatory signaling pathways. For example, both IL-1β and TNFα induce HA production in endothelial cells in an NF-κB-dependent manner (87). We have also demonstrated that HA synthesis is enhanced in tumor cells through an IL-6/STAT3-dependent mechanism (88). Furthermore, inflammatory macrophages express hyaluronidases (89) and ROS (58), which potentially fragment HA into pro-inflammatory polymers. These results predict that HA and pro-inflammatory cytokines act reciprocally to sustain inflammation.

## Contributions of Hyaluronan Receptors and Binding Proteins to Inflammation

A major challenge in the mechanistic understanding of HA in breast cancer-associated inflammation is to link HA metabolism with specific contributions of HA receptors CD44, RHAMM, and LYVE-1, which are all expressed by macrophages (78, 90–92). CD44 has been examined for its ability to regulate macrophage migration and phagocytosis (93). In the context of modulating macrophage responses to tumor cells, functional studies demonstrate a link between CD44:HA binding and generation of immunosuppressive macrophages. Specifically, blocking the ability of HA to bind to monocytes either through blocking HA:CD44 binding or using an HA-specific blocking peptide inhibits tumor cell conditioned media promoted formation of immunosuppressive macrophages (78).

While RHAMM has not been examined specifically in the context of tumor-associated macrophages, recent studies have started to elucidate its potential functions during response to injury. RHAMM expression is induced in macrophages following chemically induced lung injury (94) and in excisional skin wounds (34) and blocking RHAMM function in these injuries reduces the level of tissue macrophages (31, 34). Additional studies have demonstrated that RHAMM regulates macrophage chemotaxis in response to TGFβ in the context of surfactant protein A-mediated inflammation in the lung (92). While not specifically addressed, these studies predict the potential of RHAMM for promoting HA-mediated macrophage motility and chemotaxis in tumor-associated inflammation.

While the contributions of HA interactions with LYVE-1 to macrophage functions are even less well understood, recent interest in LYVE-1 as a marker of tumor-associated macrophages suggests that further studies of these interactions are warranted (90). Given the numerous effects of HA on macrophage recruitment and function, a focus on the roles of HA receptors in mediating tumor-associated macrophage functions will likely dramatically increase understanding of the mechanisms driving macrophages to promote breast tumor progression.

Studies have also suggested a link between HA and toll-like receptor (TLR) signaling in macrophages (27, 95). Specifically, LMW HA induces expression of pro-inflammatory cytokines and chemokines, mediated in part by TLR2 and/or TLR4 (27, 95). Additional published studies using blocking antibodies have suggested that the TLR-mediated effects may require interactions with CD44 (96). TLR signaling has been implicated in breast cancer progression, as TLR4 is expressed at high levels on invasive breast cancer cells and knock-down of TLR4 leads to reduced cell proliferation and survival (97). *In vivo* studies have suggested that TLR4 agonists can inhibit mammary tumor metastasis (98, 99). By contrast, recent studies using a potential TLR4 agonist demonstrated enhanced survival of mice in a model of tumor resection, suggesting that the contributions of TLR4 to breast cancer progression are complex (100). Recent studies have suggested that breast cancer cell-derived exosomes modulate inflammatory cytokines in macrophages potentially involving both TLRs and CD44 (101). While direct interactions between HA and TLRs in breast cancer cells have not been established, additional studies examining HA and TLR signaling in both tumor cells and the microenvironment are warranted.

Tumor necrosis factor-stimulated gene-6 (TSG-6), which is an extracellular HA binding protein, is synthesized and secreted at sites of inflammation (102). TSG-6 binds HA with high affinity via a link module and enhances binding of HA to CD44 (103). TSG-6 also contributes to HA cross-linking, which has been implicated in adhesion and rolling of leukocytes (104). In the context of breast cancer, TSG-6 is up-regulated in breast cancer cells following ionizing radiation, suggesting a potential role for TSG-6 when tissue is damaged (105). It will be interesting to determine the contributions of TSG-6 to HA remodeling and function within the breast cancer microenvironment.

## Effects of Hyaluronan on Adaptive Immune Cells in Cancerized Stroma

In addition to innate immune cells, adaptive immune cells are also prevalent within the breast cancer microenvironment. Immune cell profiling studies have demonstrated that breast cancer with high levels of macrophages and Th2 T cells are associated with worse outcome than those with high levels of Th1 cells (106). More recently, studies have demonstrated that the presence of infiltrating T cells and B cells predict better response to neo-adjuvant chemotherapy in breast cancer patients (107). Understanding the regulation and function of adaptive immune cells during both tumor progression and therapy is a rapidly growing focus of research in the breast cancer field.

While the potential role of HA on tumor infiltrating lymphocytes has not to our knowledge been reported, HA is known to contribute to the regulation of T cell trafficking (Figure 2). Studies have demonstrated that upon activation, T cells adhere to and migrate on native HA (108). Other studies show that HA:CD44 interactions on T cells can contribute to activation-induced T cell death (109). This response occurs following exposure to HMW, rather than LMW HA, suggesting an additional anti-inflammatory role for HMW HA. Finally, HMW HA has also been found to promote the immunosuppressive functions of regulatory T cells (Tregs) (110). Exposure of Tregs to HMW HA leads to prolonged expression of Foxp3, a transcription factor that is required for Treg function. Collectively, these studies predict an important role for HA in the regulation of T cell recruitment and/or function.

## Targeting HA Metabolism as a Potential Therapeutic Strategy in Breast Cancer

Given these links of HA and its receptors with breast cancer progression, targeting HA metabolism represents a potential therapeutic approach for treatment of breast and other cancers. There are multiple potential points in the HA metabolic pathway that could potentially be targeted including HA synthesis, accumulation, degradation, and/or HA:receptor interaction. Use of 4-MU, an inhibitor of HA synthesis, is a common approach for blocking HA synthesis in experimental models of breast cancer and is described in detail in another article in this Research Topic (111). Numerous studies have demonstrated that inhibition of HA synthesis using 4-MU reduces breast cancer tumor cell proliferation and migration (88, 112, 113). Furthermore, treatment of tumor bearing mice with 4-MU reduces tumor growth (114, 115). Treatment of mice bearing bone metastatic lesions with 4-MU reduces HA accumulation and growth of osteolytic lesions (116, 117). 4-MU is well-tolerated in both animal models suggesting that blocking HAS catalytic function represents a viable therapeutic strategy. While the efficacy of targeting HA synthesis alone remains to be determined in human cancers, we have recently demonstrated that reducing HA synthesis combined with targeted therapy enhances therapeutic response (88). These studies highlight the importance of combinatorial targeting of both tumor cell specific oncogenic signaling pathways and pro-tumorigenic alterations in the tumor microenvironment in new therapeutic approaches.

Elimination of HA in the tumor microenvironment using hyaluronidases has also been explored as a potential therapeutic strategy for some cancers, including pancreatic cancer, and is currently being tested in clinical trials (118–120). Treatment of breast cancer cells with bacteriophage hyaluronidase inhibits growth, migration, and invasion in culture (121). Recombinant hyaluronidase, which eliminates stromal HA, allows increased drug access to tumor cells (118–120). Studies suggest that recombinant human hyaluronidase (rHuPH20) improves subcutaneous delivery of antibody-based targeted therapies such as trastuzumab, currently used for treatment of HER2-positive breast cancer (122). HA is a normal component of the breast stroma that provides structural support and contributes to epithelial morphogenesis (123). Whether eradication of HA and/or the generation of fragments due to the hyaluronidase activity negatively affects breast tissue architecture remains to be determined.

Additional approaches to inhibiting HA function in tumors include interfering with HA:receptor interactions. CD44 expression correlates with specific subtypes of breast cancer, including triple negative and endocrine resistant breast cancers (124, 125). Furthermore, HA–CD44 interactions promote invasion and therapeutic resistance (7, 124, 125). Thus, developing targeted therapies that specifically inhibit this interaction could lead to viable therapies for treating breast cancer subtypes that currently have limited therapeutic options. Nevertheless, the use of a humanized monoclonal antibody (Bivatuzumab) in clinical trials of patients with squamous cell carcinomas showed early promise. However, it had a dose related toxicity in some patients and caused the death of one patient causing the trial to be terminated prematurely (126) raising concerns about this therapeutic approach. Furthermore, since there are multiple structural variants of CD44, it may be difficult to develop a complete array of humanized antibodies that can target this structurally complex group of proteins.

An alternative approach, which may be less toxic than Bivatuzumab will be to develop and utilize HA binding peptides that can specifically block HA-stimulated signaling and inflammation. Early efforts along this line using a 12mer phage display resulted in a peptide termed PEP-1, which was identified by sequential binding of 12mer-displaying phage to immobilized HA (127). PEP-1 has been shown to reduce gastric stem cell proliferation (128) and reduce *H. pylori*-induced gastric epithelial proliferation *in vivo* (128). Finally, PEP-1, in combination with the selective activation of the adenosine A2 receptor, inhibits arthritis-associated inflammation (129, 130). While the PEP-1 was effective in these studies, it was not demonstrated to inhibit interactions with a specific HA receptor. More recently, we have developed a unique HA binding “RHAMM mimetic” peptide using a 15mer (P-15) based phage display approach (34). This 15mer approach is unique from PEP-1 in several respects. Unlike PEP-1, P-15 contains a BX_7_B HA binding motif found in RHAMM, it binds HA, in particular HA fragments with high affinity, can inhibit HA binding to RHAMM but does not block HA binding to CD44. It inhibits HA-stimulated migration of RHAMM^+/+^ fibroblasts but has no effect on the migration of RHAMM null fibroblasts. P-15 reduces inflammation, angiogenesis, and fibroplasia of RHAMM^+/+^ but not RHAMM^−/−^ excisional wounds. Peptides or mimetics similar to P-15 may offer an effective alternative therapy since specific blockade of RHAMM can also limit CD44 signaling.

## Summary

In summary, there is clear evidence that alterations in HA are associated with malignant progression of breast cancer. Based on the known pro-inflammatory properties of HA fragments during wound healing and the increased levels of HA associated with the peri-tumor stroma in breast cancers, it is likely that HA contributes to the generation of a pro-tumorigenic inflammatory environment. This is supported by the recently identified links between HA levels in the tumor stroma and infiltration of macrophages. Analyzing the presence and function of HA fragments within the tumor microenvironment will provide insights into changes in HA metabolism during tumor growth and progression. As described in an accompanying article in this issue (36), advances have been made in the isolation of HA from tissues and analysis of HA fragmentation and addressing these questions is now feasible. Identifying the specific HA receptors involved in mediating recruitment and activation of inflammatory cells, such as macrophages, into the tumor environment and determining how HA regulates adaptive immune cells will lead to a better understanding of how alterations in HA contribute to host immune responses to breast cancer. Agents that limit aberrant HA synthesis, fragmentation, or block specific HA:receptor interactions is very likely to yield advances in the development of new therapies to limit relapse and recurrence in patients receiving tumor cell targeted therapies.

## Author Contributions

KS, MC, PT, ET, and JM contributed to the drafting and revising of this manuscript. All authors approved this manuscript.

## Conflict of Interest Statement

The authors declare that the research was conducted in the absence of any commercial or financial relationships that could be construed as a potential conflict of interest.
